# Predicting Divine Action

**DOI:** 10.1007/s11406-018-9947-z

**Published:** 2018-02-15

**Authors:** Hugh Burling

**Affiliations:** St John’s College, University of Cambridge, Cambridge, CB2 1TP UK

**Keywords:** Theism, Natural theology, Philosophical theology, Swinburne, Divine action, Skeptical theism

## Abstract

This article sets out a formal procedure for determining the probability that God would do a specified action, using our moral knowledge and understanding God as a perfect being. To motivate developing the procedure I show how natural theology – design arguments, the problems of evil and divine hiddenness, and the treatment of miracles and religious experiences as evidence for claims about God – routinely appeals to judgments involving these probabilities. To set out the procedure, I describe a decision-theoretic model for practical reasoning which is deontological so as to appeal to theists, but is designed not to presuppose any substantive moral commitments, and to accommodate normative and non-normative uncertainty. Then I explain how judgments about what we probably ought to do can be transformed into judgments about what God would probably do. Then I show the usefulness of the procedure by describing how it can help structure discussions in natural theology and a-theology, and how it offers an attractive alternative to ‘skeptical theism’.

## Introduction

This article sets out a formal procedure for determining the probability that God would do a specified action, using a subset of the theologian’s beliefs I will refer to as ‘moral knowledge’: our beliefs about what is right for us to do, and our beliefs about the details of situations which bear on what we should do. Why would we want to determine such probabilities? To begin answering, I point out how predictions of divine action are central to natural theology, as well as relevant to other pieces of religious reasoning (Section 2). After setting out the procedure (Sections 3–6), I will give a better answer to another obvious criticism. Perhaps natural theology has got by well enough so far with implicit or explicit judgments about what God would do, and when we have needed to be explicit about the probability of these predictions, ‘ball-parked’ figures based on ‘hunches’ or ‘intuitions’ have served us well, whereas the precision I offer brings needless complexity. I will therefore explain (Section 7) how this increased precision helps interlocutors to structure discussions and focus on the deeper sources of disagreement in natural theological controversies. Since my procedure is built around a decision-theoretic model for moral reasoning, one would expect technical problems to arise from the difference between our knowledge and power, and God’s. Accordingly, there may be ways in which my procedure can be embellished or improved upon, both piecemeal and wholesale. The one I set out here is really intended to show that this increased precision in natural theology is a realistic and worthwhile goal. Throughout, therefore, I will indicate suggestions for modifications in footnotes.^1^

## Divine Action Prediction in Natural Theology

Natural theology involves, explicitly or otherwise, a great many claims about what God would do: that is, predictions of divine action. Natural theology^2^ which operates in a probabilistic framework – arguing that some phenomena independent of revelation confirm, or disconfirm, God’s existence – thereby involves a great many claims about what God would *probably* do. Here are some examples.

- *Design Arguments*: Anyone who thinks that the improbability of humans existing on naturalism – given, say knowledge about cosmological fine-tuning – confirms theism must believe something like “it is not surprising or improbable that God would create a world that could support intelligent life” (Collins 1999, 54) or, more precisely, “there would be a probability of half [or, at minimum, greater than the probability that humans would emerge on naturalism] that God will bring about some humans” (Swinburne 2012, 108). Among projects within natural theology, design arguments are particularly conducive to explicit formulation in terms referring to the probability that God would do something or other.

There are arguments for and against God’s existence which are more forcefully presented if they describe their prediction of divine actions as ‘analytic truths’. In these cases, probability only enters the picture when we’re considering whether the phenomena under consideration have really occurred. Here are some examples, and why they really involve predictions about divine action.

- *‘Gratuitous’ Evils*: If someone thinks that the amount of suffering we see in the world disconfirms theism, they must believe something like the two premises of Rowe’s influential evidential argument from evil: “2. [God] would prevent the occurrence of any intense suffering [He]^3^ could, unless [He] could not do so without thereby losing some greater good or permitting some evil equally bad or worse” in addition to “1. There exist instances of intense suffering [of the above kind]” (Rowe 1979, 336). But if we have evidence for theism, Rowe’s second premise implies only that his first premise is false. So an evidential argument from evil needs to refer to some individual occurrence of suffering; then, by feeding an account of the intensity and global worthlessness of that suffering^4^ into the account of God’s decision-making which Rowe’s second premise implies, predict that ‘God would prevent this suffering’.^5^ Clearly, however, theists can contest such predictions: it seems appropriate to insert a ‘probably’ into them. Theists’ reasons for doubting such predictions will typically have to do with their disagreement about the goods or obligations at stake: perhaps they have an appropriate theodicy.

- *Divine Hiddenness*: If someone thinks that lack of belief in God’s existence disconfirms theism, then they typically base this argument on predictions of divine actions such as the following: “God would desire a reciprocal personal relationship between himself and every being capable of it” (Wainwright 2002, 98) and “God would put his existence beyond reasonable nonbelief” (Schellenberg 1993, 3). Conversely, those who don’t think these phenomena significantly disconfirm theism tend to base their arguments around predictions such as “God would not force himself upon us…[but] would leave it up to us to enter into [a loving relationship with Him]” (Howard-Snyder and Moser 2002, 13) or “God would [refuse] to bring people to belief unless they were sincerely open to [a certain sort of] moral transformation” (Howard-Snyder and Moser 2002, 16–17, paraphrasing Moser 2002). As with evidential arguments from evil, the extent to which divine hiddenness phenomena confirm or disconfirm theism will depend on the probability observers assign to these predictions.

- *Arguments from miracles, and evaluating the miraculous character of extraordinary events*: General arguments from the occurrence of miracles to theism are typically presented as probabilistic, and sometimes explicitly refer to the probability of some miracle given theism (e.g. Holder 1998, 59–63). As with design arguments, the feature of theism which is going to make probable the occurrence of the miracle will be whether or not God would bring about that extraordinary event: that is, a prediction of a particular divine action.^6^ But when we are evaluating competing claims about God which refer to putative miracles as evidence to support them, we will be evaluating competing predictions of divine action. Loosely put, someone might think that God is more likely to become incarnate than reveal the Koran, and so think that the former is more likely to have occurred than the latter.

- *Arguments from religious experience and evaluating claims to veridical religious experiences*: An inductive argument from the general occurrence of religious experiences requires theism to predict the occurrence of religious experiences more than atheism does. An inductive theological argument that God has some particular feature, from some set of appropriate religious experiences, requires the more particular prediction that God would give the veridical religious experiences testifying to that feature. When it comes to the more quotidian problem of evaluating particular claims to religious experience, we might first check that the ‘candidate mystic’ is a reliable testifier in general terms. Then, perhaps, we’ll evaluate their experience-supported claims in terms of coherence given other background knowledge we have about God (such as information coming from our religious tradition). But, in addition, “there may be arguments to show that it is very, very improbable that…God…would have said or done what the subject claims to have experienced him saying or doing” (Swinburne 2004, 324).

Practitioners of natural theology routinely rely upon predictions of divine action. And there is a great deal of disagreement between them: most notably, between atheists and theists. Perhaps much of that disagreement can be traced back to the different probabilities they assign to divine actions. In that case, to help resolve such disagreements fairly, they will want a procedure for determining what those assignations should be given their other beliefs. Apart from this, there is the question of the truth about God’s existence and activities, and natural theology as a discipline which seeks that truth. If probabilistic inferences preserve truth, then our procedure will be helpful in discovering or confirming whether or not God exists, and what He is likely to do or have done.

## How Perfect Being Theology Generates “Anselmian Auguries”

To help us with natural theological reasoning, we want a procedure that will allow us to determine the value of the epistemic probability that God will carry out a specified action: henceforth I’ll use the placeholder *Gw* to refer to any specified divine action, so P(*Gw*) is the probability that *Gw* (would) occur(s), that is, that God would so act. But the probability that *Gw* occurs given what other information? I suggest that the most proximate and general beliefs we should look to are beliefs about God’s obligations, that is, beliefs about what an omnipotent being would do, supposing they are morally unsurpassable. I will write as though God has a complex, active mental life, and is subject to and can satisfy obligations. This can all be construed as metaphorically or analogously as our accounts of the divine mind, divine goodness, or theological predication require, since the account of obligations I give in due course is intended to generalize between deontological, consequentialist and virtues-based accounts of ethics.

We can divide a perfect being’s perfections into those which relate to His character, and those which relate to His capabilities. Among the latter will fall features such as omnipotence and omniscience. Independently of His intentions, nothing about these latter features imply that He will carry out some particular action, but only remove limits on which actions are available to Him. If, however, we believe something about God’s character, then we might find aspects of it which would motivate Him to choose one course of action over another. If God is a morally unsurpassable agent, then this sets a constraint on which might guide His actions: God will only act on good intentions (or reasons, or motivations, etc.); the better some intention relative to other good intentions, the more important a determining factor it will be for God when He is deciding how to act. So we can work out what God’s actions would probably be by considering our moral intuitions about the probity of various candidate divine intentions. There’s no reason, however, for prioritizing talk of ‘good intentions’ when considering what actions a perfect being is likely to take. There’s a whole range of alternative semantics: a morally unsurpassable being will always satisfy their obligations, manifest as much of every virtue as possible in each virtue-manifestation opportunity, maximize the occurrence of good consequences, and so on.^7^ Whatever the right-making features of a right action are, all God’s actions will have them. Any of His action-guiding mental states will be characterized in terms of them.

Indeed, when we return to the specific predictions of divine action quoted in the discussion of natural theology above, we always find references to God’s power and knowledge used to ‘remove excuses’, but references to God’s morally unsurpassable character used to defend the claim that God would do the action in question: I’ll re-use our two divine hiddenness theodicies as concise examples: “God would not force himself upon us, *as that would make the relationship a sham*. [Therefore] He would leave it up to us to enter into [a loving relationship with Him]” (Howard-Snyder and Moser 2002, 13, my emphasis). And, “God would manifest *His perfect love* by refusing to bring people to belief unless they were sincerely open to [a certain sort of] moral transformation” (Howard-Snyder and Moser 2002, 16–17, my emphasis). Our beliefs about right and wrong, both in terms of quite general principles, such as preventing unnecessary suffering as in Rowe’s evidential argument, and in terms of quite fine-grained judgments, such as claims about what sort of activities the best interpersonal relationships involve, provide the evidence for predictions of divine action and hence the resources for natural theology.

The assumption that God is to be defined as a perfect being is strongly associated with St Anselm: so belief in the object of perfect being theology is sometimes called ‘Anselmian theism’. And this puts us in a position to give a name to our formal procedure, for ease of reference. In the ancient days when we were subject to the elements of the world, their worshippers attempted to predict their actions by carrying out procedures called “auguries”. So I suggest we name any procedure we use to predict the behaviour of a perfect being using our moral knowledge an “Anselmian augury”.^8^ My suggestion for how to carry out an Anselmian augury comes in three parts: laying out a set of terms for describing moral reasons which is independent of any moral theory (Section 4), adapting standard decision theory to show how we should weigh our moral reasons, so described (Section 5) and showing how this relates to God’s actions (Section 6).

## The Deontic Discourse

Insofar as reasons for action salient according to one moral theory can be paraphrased into reasons salient in another, we need not determine which moral theory is the ‘correct’ or ‘most fundamental’, and which of the alternatives are mere conveniences, for our natural-theological purposes. In this section, I outline a deontological account. Why use a deontological account, rather than sticking with consequentialist moral decision-making procedures in which we weigh the goods putatively produced by different actions?^9^ Firstly, many theists, and hence many natural theologians, aren’t friendly to consequentialism, and so using the deontic discourse will hopefully prevent the propriety of Anselmian auguries from being held hostage to a moral-theoretic position many would-be augurs reject. Secondly, consequentialist accounts of moral decision-making are more beholden to problems arising from infinitarian utility aggregation (e.g. Bostrom 2011), problems which in God’s case cannot be dealt with by reference to His ignorance or limited causal power.

Here’s a suggestion of how to carry out the paraphrasing: call this the ‘deontic discourse’.

Call a *subjective obligation* whatever action one ought to do given one’s circumstances and knowledge, including moral beliefs, beliefs about moral theories, and everything else ‘introspectively accessible’. One’s subjective obligation will be what one honestly judges is the right particular action to take, given one’s information.

We will have subjective obligations whatever moral theory we subscribe to and whatever our particular beliefs about what the goods, *pro tanto* duties, good intentions, or virtues are. If you are an act utilitarian and, given your beliefs, you think the action which will maximize happiness right now is to make some tea, then you are subjectively obliged to make some tea.

Your *sans-phrase obligation*^10^ is the action which you aim to discover when you carry out situation-specific moral reasoning. Supposing that one can’t be blamed for doing one’s best, and how well one can do is determined by one’s moral (*inter alia*) knowledge, one can’t be blamed for carrying out one’s subjective obligation instead of one’s *sans-phrase* obligation. But *sans-phrase* obligations have to be on the conceptual table as the goal towards which practical reasoning aims.

Suppose you’re an act utilitarian, and act utilitarianism is true, and you think that the way to maximize happiness is to make some tea, but unbeknownst to you (your colleagues are too polite) making coffee would generate more happiness. Then making coffee would be your *sans-phrase* obligation; you can’t be blamed for not doing it; in a way you can’t be required specifically to make coffee rather than tea, given your ignorance. Suppose you’re an act utilitarian, and you believe making tea will maximize utility, and not only are you wrong about that, but act utilitarianism is false – Nietzschean virtue theory is true instead. As a consequence your *sans-phrase* obligation is to manifest your will to power by commanding a colleague to bring you wine. Your decadent utilitarian beliefs are sufficient to ensure your failure to satisfy your *sans-phrase* obligation, but given that you had them you still couldn’t have done better, so you’ve satisfied your subjective obligation.

A *pro tanto* obligation is an obligation which you must satisfy whenever possible, and which can be satisfied through a range of particular actions. *Sans-phrase* obligations are determined by the circumstances an agent is in and which *pro tanto* obligations those circumstances make it possible for us to satisfy. In our example above, if act utilitarianism is true, there’s just one *pro tanto* obligation, the obligation to maximize happiness. In that situation I can satisfy happiness more by making the coffee than by making tea, waiting for another to do it, or leaving it undone, so I’m *sans-phrase* obliged to make coffee. If I’m an act utilitarian and I am aware that this is the way to maximize happiness, I’ll also be subjectively obliged to do it.

On many other moral theories, however, agents are subject to more than one *pro tanto* obligation, and they must ‘balance’ these in circumstances where the opportunity to satisfy multiple *pro tanto* obligations arises, but it is impossible to satisfy all of them. As we saw with the utilitarian case, any high-level moral theory can be trivially paraphrased in terms of *pro tanto*, *sans-phrase* and *subjective* obligations. We might say that if Kant is right we are *pro tanto* obliged to act only on a (set of) special maxim(s); if Aristotle, we have a *pro tanto* obligation to cultivate (or manifest) each of our ‘virtues’, special character traits.

Note well that in the deontic discourse we are never under conflicting *sans-phrase* or subjective obligations. ‘Moral dilemmas’ are only generated by tension between *pro tanto* obligations. Whenever it looks as though there are two different courses of action which are different ways of satisfying the same *pro tanto* obligation, but we think one of those might be the better course of action than the other, what is really going on is that the two courses of action allow us to satisfy different degrees or combinations of *pro tanto* obligations.

## Balancing One’s *Pro Tanto* Obligations

For the sake of concision, I will present the following procedure for attempting to determine one’s *sans-phrase*, and thereby determining one’s subjective, obligation, but not fully defend it. In presenting the procedure I aim to show it ‘saves the appearances’ of our ordinary moral intuitions.^11^

According to the deontic discourse, whenever I am presented, by my circumstances, with an opportunity to satisfy one *pro tanto* obligation by doing some action within my power, I’m *sans-phrase* obliged to do that action. I’m subjectively obliged to do that action which, given my moral beliefs and knowledge of my powers and the features of the situation, seems to be my *sans-phrase* obligation. If my circumstances present me with the opportunity to satisfy more than one *pro tanto* obligation, however, and I can’t satisfy both, I must ‘weigh’ them against each other in order to decide which to satisfy – to decide, that is, which particular action I ought to do here and now. Suppose that only by doing a particular action *x* can I satisfy one *pro tanto* obligation, *Ox*, but only by *y*-ing can I satisfy another of my *pro tanto* obligations, *Oy*. I am in a situation where I know I can do either *x* or *y* and know *x* will satisfy *Ox* and *y* will satisfy *Oy*. I must weigh *Ox* and *Oy* against each other to determine whether I ought to *x* or *y*.

What are we weighing here? Call the term of measurement by which the two actions are commensurable their *rightness*. Our *sans-phrase* obligation is to do the action which, of the alternatives, has the most rightness. But because of uncertainty about whether, which and how far any action will satisfy a *pro tanto* obligation, our subjective obligation is the action which, of the alternatives, has the highest *expected rightness* (ER), which is determined in the following way.^12^

I’ll describe how to calculate the expected rightness of an action by fixing as many variables at first, and describing how to accommodate change in those variables as we progress.

We are in a situation where we can *x* or *y*, but not both *x* and *y*. Suppose that we have credence 1 that action *x* will satisfy *pro tanto* obligation *Ox*, but not satisfy *pro tanto* obligation *Oy*, and *y* will satisfy *pro tanto* obligation *Oy* but not *Ox*. Suppose *Ox* and *Oy* can’t be satisfied to greater or lesser degrees. We want to know whether to *x* or *y*. Either *x*ing and *y*ing are on a par (Chang 2002),^13^ equal, or one has higher expected rightness than the other. If they are on a par or equal, it’s permissible to either *x* or *y*. Suppose that the third possibility is the case and *x* has greater ER than *y*. In virtue of what would it have this? It would have to be that satisfying *Ox* bears more rightness than satisfying *Oy*.^14^ We can think of this relative rightness-bearing of different *pro tanto* obligations as the ‘strength’ with which different *pro tanto* obligations ‘bind’ moral agents. Now, if *Ox* is stronger than *Oy*, they must both occupy positions on a scale of obligation strengths. In due course we’ll look at how to reflect on relative obligation strengths; for now, we’ll represent the obligation strengths of *Ox* and *Oy* by *n* and *m* respectively.

Unfortunately, however, we might be uncertain about the claims that (*p*) “*x* will satisfy *Ox*”, (*q*) “*y* will satisfy *Oy*”, (*p*’) “*Ox* is *n* strong”, and (*q’*) “*Oy* is *m* strong”. Those uncertainties – conversely, those credences in, or the subjective probabilities we assign to, *p*, *q*, *p’*, and *q’*, will make a difference in our attempt to investigate our *sans-phrase* obligation, by changing the ER of *x* and *y*.

Let’s worry about the effects of ‘normative uncertainty’ (Sepielli 2009, 5), or credences lower than 1 in beliefs like *p’* and *q’*, first. I’ll be less sure that *x*, not *y*, is the right action insofar as I’m unsure that *Ox* is stronger than *Oy*, but also due to the differences between the strengths of *Ox* and *Oy* about which I’m unsure. Consider a case in which I’m unsure whether saving a life is more important than telling the truth. Whether I think that I ought to lie to save a life will depend not just on how sure I am that saving lives is more important than telling truths, but also on how much more important than telling truths I think saving lives is. The relationship between these two factors can be expressed by a single value – let’s call it an ‘obligation credence’ – which we determine by treating our credences in *p’* and *q’* (call these *Cp’* and *Cq’*) as probabilities that *p’* and *q’* are true. If we ‘just knew’ *p’* and *q’* then we would ‘know’ that satisfying *Ox* will have a rightness tracking *n* and satisfying *Oy* will have a rightness tracking *m*. Because we don’t, however, satisfying *Ox* has an ER equal to *n* multiplied by the probability of (i.e. our credence in) the truth of *p’*; satisfying *Oy* has an ER equal to *m* multiplied by the probability of the truth of *q*’. Call the obligation credences for *Ox* and *Oy,* ‘*OCx*’ and ‘*OCy*’, and think of them as *pro tanto* expected rightnesses for satisfying the *pro tanto* obligations, wherever, whenever. The values for *OCx* and *OCy* can then be used to ensure the procedure as a whole accounts for normative uncertainties.^15^

‘Non-normative’ uncertainties (Sepielli 2009, 5), credences below 1 in propositions like *p* and *q*, also play a role in calculating the ER for particular actions. If I think it sufficiently unlikely that telling this lie will save this life, I will think it wrong to tell the lie even though I think life-saving is much more important than truth-telling. Call the credences concerning propositions like *p* and *q* our ‘satisfaction credences’. Now that we have obligation credences and satisfaction credences, we can calculate the ER of an action in a way which accounts for normative uncertainty, non-normative uncertainty, and the differences in strengths between different *pro tanto* obligations. Just multiply the satisfaction credence for an act by the sum of the obligation credences for all the *pro tanto* obligations one believes that act has a chance of satisfying. (If we like, we can split up the satisfaction credence for the act into a set of satisfaction credences, one for each *pro tanto* obligation we think the act might satisfy, and add those together, then multiply the result by the sum of the obligation credences for all those *pro tanto* obligations.)^16^ Note that when this procedure is for comparing mutually exclusive courses of action. Multiple courses of action which can be carried out together can be combined into one.

Three popular moral intuitions are not accounted for yet.

Firstly, it sometimes seems as though *pro tanto* obligations can be satisfied to greater or lesser degrees – consider, for example, beneficence. I suggest that any *pro tanto* obligation which admits of degrees of satisfaction be treated as a set of *pro tanto* obligations, one corresponding to each degree. The member of that set relevant to calculating the ER of an action is the one corresponding to the degree of satisfaction that action is expected to reach.

Secondly, it sometimes seems as though some *pro tanto* obligations are stronger than others, even though these others might be capable of being satisfied to infinite degrees. Consider some peoples’ intuitive rejection of the torture option in ticking time-bomb cases: some think we’re obliged not to torture, however many lives are at stake.

This intuition seems to suggest there might be *pro tanto* obligations with infinite strength. That infinite strength will swamp the significance of the satisfaction credences regarding any actions we think might satisfy an infinitely strong *pro tanto* obligation. We’ll always be subjectively obliged to undertake any course of action with any chance at all of satisfying any infinitely strong obligations.

One possible solution is to install a Sure Loss Principle^17^ into the procedure to prevent the various bizarre effects of putative infinite expected rightnesses. Unfortunately, this solution doesn’t save the appearance of the intuition that there are infinitely strong *pro tanto* obligations. Sure Loss Principles tell us to forego low chances to get great goods when the associated cost exceeds some topic-neutral threshold. Such a threshold would have the effect of treating the infinitarian obligation as though there were a ‘cap’ on its relative strength. I take this consequence to indicate that our intuitions about infinitely strong *pro tanto* obligations are unreliable. How we should model a *pro tanto* obligation advertised as infinitely strong is to ask its advertiser to consider expensive courses of action with very low probabilities of satisfying the obligation in question. Keep reducing the probabilities while holding the expenses stable: when they reject the expense, they have assigned that obligation a finite strength.

The third ordinary moral intuition we should accommodate concerns the wide scope of situations in which it seems permissible, even obligatory, for agents to act in pursuit of ends they have chosen for themselves, because they have chosen them for themselves. No-one is *pro tanto* obliged to become a philosopher, just *qua* moral agent. But if I have set my heart on it, perhaps I am thereby so obliged, so that it would be right for me to forego actions otherwise binding on all. If this is true, it implies the existence of situations in which, among a range of actions with equal highest ER, some are more rational to pursue than others: those actions which pursue the agent’s idiosyncratic ends. We can treat pursuing one’s own ends as a *pro tanto* obligation and work out its background ranking against other *pro tanto* obligations which bind all agents equally.

Finally, let’s consider how to assign obligation strengths, as promised above. Andrew Sepielli’s solution is to rank *pro tanto* obligations cardinally, since in normal reasoning cases the differences in strengths will have to be significant enough to ‘bear up’ on multiplication with credences, so that it isn’t the credence which does all the work (Sepielli 2009, 10–20). To show the plausibility of this strategy, he appeals to “a good old-fashioned trolley problem” (Sepielli 2009, 17). If you believe that it’s worth letting (at most) five people die in order to avoid killing one person, then you believe that the obligation not to kill is (at most) five times stronger than the obligation not to let people die. To cut a long story short, one could run trolley problems with all sorts of different consequences on the two different tracks, ensure consistency over one’s rankings, and thereby rank all the *pro tanto* obligations one recognizes.^18^ Since we want to have figures for our obligation strengths which can represent consistent ‘background rankings’ of all *pro tanto* obligations against each other from one occasion of practical reasoning to another, I propose that we assign 100 points of obligation strength to those *pro tanto* obligations which are ranked equally highest. Then assign fractions of 100 to other *pro tanto* obligations in accordance with their cardinal ranking relative to the strongest equal obligations. (So, for example, if we assigned 100 points to the obligation to not kill anyone, then the obligation not to let someone die would have 20 points of strength.) To account for obligations which admit of different degrees of satisfaction, take a *pro tanto* obligation which admits of varying degrees of satisfaction (in practice, a set of *pro tanto* obligations each of which contains a clause corresponding to each degree of satisfaction). Find another *pro tanto* obligation (either one which admits of no degrees of satisfaction, or one which is a member of one of these satisfaction-degree-indexed sets and has been ranked and assigned a value already) which is ranked equal with satisfying the former to the lowest degree. Then equate the points value of satisfying one degree of the former, degreed obligation with the points value of satisfying the latter.

A feature of this procedure, which will be important when we apply it to predicting divine action, is that it not only generates a judgment as to which particular action of a range of actions is our subjective obligation, but also generates a degree of (subjective) probability associated with each action: ‘the best’ action is really ‘most probably the best, all things considered’ action, and all the probabilities in question will be on display in the rightmost column of a decision box, where we fill in the ER of each action.

## From Moral Decisions to Anselmian Auguries

If God is morally unsurpassable and omniscient, He’ll always do whatever He’s *sans-phrase* obliged to do.^19^ Failing to do what we are *sans-phrase* obliged to do is only possible for us either because of our ability to wilfully sin, or because of our ignorance, which leads to discrepancies between our subjective and *sans-phrase* obligations. But when we try to work out what God is *sans-phrase* obliged to do, we must ‘put ourselves in God’s place’ using only our imperfect moral knowledge, with all its uncertainty, and so we get a judgment only about what our *sans*-*phrase* probably is, our ‘subjective obligation’. So the procedure for determining our subjective obligation is the procedure for determining what God would probably do, whenever the decision to be made is one we think God must make or has made.

Perhaps articulating the possibility of probabilistically predicting divine action sounds surprising or impious. But scepticism about our ability to predict divine action using perfect being inferences is easily explained by our awareness of the normative and non-normative uncertainty we live under. We are rightly hesitant to make claims of the sort “this would be the right action for an omnipotent being, so God will do this”. But theologians do make judgments of that sort all the time: what we want to ensure is that those judgments reflect our moral uncertainty. The Anselmian augury ensures our judgments have this feature; that theological doubt tracks moral doubt. So those worried about risk of hubris and error in natural theology should welcome, not resist, procedures like the augury. Here is how the augury translates judgments about the ER of putative mutually exclusive divine actions into probability values for each of those actions.

God’s omnipotence and eternity mean that in any perceived or conceived creaturely circumstance which provides Him an opportunity to satisfy one or more of His *pro tanto* obligations, He will be able to, and so will be *sans-phrase* obliged to, and hence will, perform the action determined as optimal in that circumstance by our weighing procedure. Or, rather, we will think it probable that He will in exact proportion to how likely we think this the best action, given the procedure’s inputs. The action with the highest ER among the outputs of the weighing procedure where we ‘put ourselves in God’s place’ will be the one He is most likely to do.

For step one, we describe some morally salient feature of the world in a proposition, or set of propositions, which suggests clearly enough what good is missing from or present in the scene, in such a way that it’s clear enough which *pro tanto* obligations would be satisfied by God acting in that circumstance. These are one step from the propositions which take our satisfaction credences. The way in which God’s case works differently from the creaturely case at this step is as follows. The propositions which take our satisfaction credences when we are engaged in practical reasoning typically refer explicitly to some action by which we might satisfy the relevant obligations. In our case, our credence reflects our uncertainty as to whether that action will in fact satisfy those obligations. In God’s case, however, His actions don’t need any specific descriptions – wherever He turns His ‘hand’, He just brings things about in such a way that He’s satisfied His *sans-phrase* obligation. So when we ‘put ourselves in God’s place’, our satisfaction credence reflects our uncertainty that we’ve accurately characterised the situation.

Here’s a familiar example. We might think that there’s a set of intellectually, relationally capable non-theists, and so come to believe that (*1*) “no people belonging to this set have the theism necessary to love God, and all of them might”. We can see immediately that God could satisfy His *pro tanto* obligation to be in a loving relationship (call this obligation *Oz*) by (at least) bringing it about that they believe in Him (call this action *z*). So we infer from *1* that (*2*) “*z* will (allow God to) satisfy *Oz*”. We won’t have any doubt about *2* on account of the possibility of God’s failure. Rather, any doubt we have results from, and so tracks, doubt we have in *1*.

We need a proposition of a form which can take a satisfaction credence, like *2* but not *1*: so step two is to take that proposition gave in step one, and derive a proposition like *2* which describes a course of action will satisfy one or more obligations. Then we assign a satisfaction credence to *2* equal to our credence in *1*. During step two, we might notice that God can’t, in one course of action, satisfy all the *pro tanto* obligations which bear on him in the circumstance described by the *1*-like proposition. In such cases we will need to derive, from the circumstance-description proposition in step one, a range of propositions describing the mutually exclusive courses of action which will allow God to satisfy groups of *pro tanto* obligations bearing on Him in the circumstance. We assign each of those a satisfaction credence.

Step three is to reflect on the relative strengths of the relevant *pro tanto* obligations, using the best methods of moral reflection available to us. This allows us to determine the values for *n* in propositions of the form “*Ox* is *n* strong”.

Step four is to introspect our credences in those propositions of the form “*Ox* is *n* strong”. In step five we generate our overall obligation credence for each *pro tanto* obligation bearing on God in this circumstance. Step six is to multiply the obligation credences with the satisfaction credences and so determine the ER of each mutually exclusive action ‘available’ to God.

At the end of step six we will have an ER value for each action. These represent something slightly different from what they represented when generated for the purposes of determining our subjective obligation. In our case, once we’ve determined which action has the highest ER, we can ignore the differences between that action’s expected rightness and the lower expected rightnesses of other actions: this is just our subjective obligation now. But that is because in our case the ER values account for our uncertainty as to whether each action is really our *sans-phrase* obligation. God, however, has no such uncertainty, and His subjective obligations are not determined by accounting for *our* uncertainties. He just knows and does His *sans-phrase* obligation. So each value represents the probability, relative to the other actions available to God, of God’s taking that action. But the total of all the values will be some figure depending on the strengths of all the *pro tanto* obligations which could be satisfied in that circumstance, so will fluctuate wildly rather than being a fraction of 1, that is, a numerically represented share of an event space.

Step seven, then is to add the total of the expected values for all the actions and divide 100 by it, then multiply each ER value by the product. This will give you a decimal figure for each course of action which is at all worth God’s doing, a figure which tells you the probability of God’s doing that action. At the end of step seven, we have a set of actions by which God might satisfy various situationally-relevant conjunctions of His *pro tanto* obligations, and each action has been assigned a probability that it is God’s *sans-phrase* obligation – and hence a probability that this is what God does. So, for any putative divine action *Gw*, we can treat it as an action like *x* or *y* described above, and reach a probability that it’s what God would do when He’s in the circumstance picked out by the proposition like *2*.^20^

There may be cases where God has open to Him multiple actions of equal expected rightness. When we face such situations, we can use non-moral reasons to choose between the highest-ER actions. In God’s case, then, we will need some information from outside our moral knowledge to determine the probability He does any. Perhaps in the past God has told us something about His idiosyncratic ends, and pursuing one of these would make it more likely for Him to do one of the best equal actions than the others. Without such information, however, the equal ER of the actions will determine that each of them gets an equal share of the event space.^21^

## Why Predict Divine Action?

I conclude by responding to the criticism that developing and using procedures like mine is methodologically redundant. This criticism admits that we can make justified estimations of how a perfect being would behave, and recognizes the centrality of these to natural theology. But it insists that, since we can translate our moral knowledge into a judgment of the probability that God would do some action in an informal way, there’s little value in laying out a particular way of going about this, particularly given the complexity involved in doing so.

The best response to this objection would be to conduct a survey of natural theology in analytic philosophy of religion since arguments for and against God’s existence began being explained probabilistically – or at least, perhaps, since Swinburne’s *The Existence of God* (1979). If it follows the trend I indicated in Section 2, this survey will show that disputants disagree about the probability of God’s existence because they disagree about the probability that God would do various things – permit some particular evil, make Himself known in some particular way to some particular demographic, create intelligent animals, and so on. This survey would also, I expect, show that these disagreements often persist in spite of supposed agreement about both general moral principles and the morally salient non-normative features of the world at stake. If this were so, it would indicate that something has gone wrong.

Here is one diagnosis: where two philosophers agree that some empirical claim or moral principle is ‘very probable’, ‘probable’ or ‘plausible’, but disagree on the difference this should make to theism, they are equivocating. Either the parties to disagreement mean something different by the same informal ‘benchmark’, or the parties mean different things by one word from one premise to the next in their inferences from normative to theological claims. Adopting more precise and more developed, or less precise and less cumbersome, analogues to a procedure like mine can help us pinpoint exactly where disagreements are located, and better hold each other to account. Disagreements in natural theology could arise from disagreements about the scope of some moral principle; or how strong that principle is relative to others; or, when a principle is of a very wide scope, whether we can have any confidence that it applies in that case.

If these moral disagreements are the disagreements at the heart of natural theological controversy, we should get them out in the open and focus on them, exchanging what appears to be theological disagreement for what is really moral disagreement. Or, disagreements in natural theology might be similarly reducible to disagreements about ‘non-normative’ matters to which shared principles can be applied; again, natural theologians will profit from pinning down when this is the case so that they can move into the relevant non-theological, non-normative territory.

Where different divine action predictions turn out to depend on disagreements about fundamental moral intuitions, of the sort not amenable to argument, we can clear up whether someone’s theism is rationally permissible or obligatory (on natural-theological evidence) by implementing an account of what to do in cases of moral peer disagreement. If you have a formal framework for updating on peer disagreement,^22^ two natural theologians can implement that before or after carrying out conflicting Anselmian auguries, and work out how to revise their theological beliefs in light of their moral disagreements.

Whether or not these kinds of disagreement are routinely explicit in discussion of arguments about God’s existence, it would help to have a consistent account of how they should relate to each other. Procedures like mine map how this might work: if you have this set of moral beliefs, theism might have a tonne of probability on cosmological fine-tuning, but that would be reduced by a pound given the animal pain caused by forest fires, and an ounce given ignorance of theism in medieval Siam.

Finally, I have barely addressed ‘sceptical theism’, despite the possibility that it presents another principled objection to the value of a procedure like this one. This is partly because it is difficult to describe (Rea 2014, 484–5) in a way which doesn’t either lead to moral scepticism or reduce to a claim about normative and non-normative uncertainty of the kind Anselmian auguries are designed to accommodate. But it is also because I think the procedure I’ve outlined here suggests a better alternative. I take it that by the time we have multiplied a credence that I have some *pro tanto* obligation, by the background ranking I take it to have, by the credence I have that some action of God’s will satisfy it, and then considered that action amongst all the other actions available to God with some ER, I will end up with a rather low value for P(*Gw*). Whatever I think about what God might do, I’ll have low absolute confidence that He’ll do that absent additional evidence from revelation or religious experience. On the one hand, moreover, the more local an event which is taken to be natural-theological evidence for or against theism, the lower satisfaction credences will attach to it when it has been translated through steps one and two of the augury. On the other, when we are considering general phenomena (such as the existence of animals), the inputs to the augury could well be high enough to generate significant outputs. I take it that this is just what the sceptical theist needs: the augury typically attaches low evidential significance to particular instances of horrendous evil, by generating a low value for the probability that God would prevent the horrendous evil. But since the augury takes any and all moral knowledge for its inputs, the theist who uses it cannot be accused of any inappropriate scepticism; nor does it vitiate the power of natural theology in principle.

## Footnotes

1 I would like to thank Nevin Climenhaga, Sarah Coakley, Trent Dougherty, Douglas Hedley, Hugh Mellor and Daniel Rubio for reading and commenting on drafts of this article at various stages of its development. I would also like to thank attendees of the 2015 Summer Seminar for Philosophy of Religion at the University of St Thomas in Minnesota for discussing a presentation of the model developed in this article delivered there.

2 I’ll be using the term ‘natural theology’ to cover arguments for and against God’s existence, and ‘God’ to mean, minimally, a perfect being – more and no more precisely, a morally unsurpassable omnipotent agent.

3 I use the male pronoun for God since this is what is typically used in the literature quoted; but my usage should not be taken to render my procedure reliant on any view about religious language, or about God and His sex or gender.

4 This assumes that evidential arguments from particular horrendous evils need to make use of what Wykstra has called a “noseeum inference” (Wykstra 1996, 134). Such arguments may, however, appeal to a more epistemically basic ‘seeming’. I take it that such an “alienation experience” (Draper 1991, 141) is supposed to be defeasible and disconfirm theism by some finite amount for its subject. Then predicting divine actions will be useful in bolstering or rebutting the evidence that seeming provides.

5 An alternative way of formalizing the evidential argument from evil which requires implicit claims about what God would probably do, by charging that God would be less likely to permit the evil we see than some indifferent origin for the universe, is presented by Draper (1989).

6 Alternatively, one’s moral beliefs might lead one to think theism generally predicts against miracles – see Brown (1995) for a presentation of Leibniz’ argument to this effect – and thereby that miraculous phenomena and testimonies make theism less probable – see Overall (1985) and Larmer (2004).

7 The ‘virtues’ and ‘consequentialist’ semantics here need to be parsed carefully enough to avoid the conclusion that God must create the ‘best possible world’ if He’s ‘morally unsurpassable’ since this implication might make theism impossible. See Rowe (2004, 88–150) for a representative formulation of this argument for atheism. A concise suggestion adequate to my purposes is offered by Swinburne (2012, 108).

8 This label is not intended to make the procedure reliant on any position or argument of St Anselm’s, or any interpretation of these, other than that God is a perfect being.

9 Judged entirely in terms of how far a deontological account like mine can homogenize the structures of different normative theories, I suggest it compares favourably with consequentialist attempts, such as Brown’s (2011), in coming with fewer substantive commitments. Unlike consequentialist representations, the deontic discourse takes no stand on agent-relativity (Brown 2011, 760–761) because *pro tanto* obligations can include the indexical values required by agent-centric views; no stand on satisficing (Brown 2011, 765–767) because *pro tanto* obligations regarded by advocates of supererogation as non-moral can be incorporated as moral obligations to generate the same consequences (see section 5 for an analogous case suggesting how this might work); and it can easily be tweaked to accommodate the view that some situations present us with no right action (Brown 2011, 763–5) by abandoning my stipulation (see section 5) that it’s permissible to do any of the set of actions with equal or on-a-par (expected) rightness.

10 The term is taken from Ross (1930, 19). The difference here is intended to track the distinction between ‘objective’ and ‘subjective’ or ‘external’ and ‘internal’ obligations – see Zimmerman (2008) for a thorough treatment of this – but I avoid using objective obligation here as I don’t want to imply that *pro tanto* obligations, or even subjective obligations, are not objective in the binding sense.

11 The procedure I offer simplifies Ted Lockhart’s (2000, 22–46).

12 For some accounts of why we ought to use expected value to decide what to do when we are uncertain, see Hudson (1989), Lockhart (2000, 3–46), and Sepielli (2009). I assume that we are ‘risk-neutral’ – but should we, and would God, be? It’s harder to defend a strategy for risk when the decision-maker is an omniscient, omnipotent agent. Suppose, following Buchak (2013), that multiple attitudes to risk are rationally permissible and formally expressible. Each attitude God might take would determine a different output. We can weight these outputs, as values for Pr(*Gw*), by our confidence that their attitude to risk is the wisest.

13 Let cases of parity be equivalent to cases of equality for Anselmian auguries.

14 For a more detailed defence of the idea that moral rightness comes in degrees see Lockhart (2000, 80–92).

15 Here’s the formula for assigning the obligation credence for a *pro tanto* obligation *d* with a suspected strength *s* predicated of *d* in the propositional content of the moral belief that *b*: *OCd* = *Cb***s*.

16 Here’s the formula for determining the expected rightness of a course of action, *ERx*, which cannot be performed in combination with any other actions: *ERx = ∑*^*n*^*SCa...a*_*n*_**∑*^*n*^*OCd...d*_*n*_. *SCa*_*n*_ represents the satisfaction credence for one of the propositions describing how action *x* will satisfy the corresponding one of the *pro tanto* obligations *d*. More simply, *ERx* = *SCa*∑*^*n*^*OCd...d*_*n*_, if one is ready to assign a single credence to the extended proposition *a* which describes an action as satisfying *pro tanto* obligations *d...d*_*n*_.

17 See (Jordan 2006, 115) for an easily adaptable example.

18 Moral psychologists will rightly balk at attempting to find out people’s moral beliefs in this way, citing evidence that people respond inconsistently to questions about their values – for an apposite study see Bauman et al. (2014). Improved procedures developed in moral psychology can be substituted.

19 Libertarians about divine freedom can add “freely” after “always”. That God’s actions are free in the libertarian sense will not affect the probabilities of divine actions, since those probabilities are conditional on the reasons God has for acting, not on determining conditions of the kind excluded by libertarianism.

20 And so, finally, a formula for P(*Gw*), where *x* is the action referred to in *Gw* as the action God would take, *SCa* represents the satisfaction credence for one of the propositions describing how action *x* will satisfy the corresponding one of the *pro tanto* obligations *d*_*n*_, *y* is the (set of) action(s) excluded by God’s undertaking *x*, *SCb* the satisfaction credence for one of the propositions describing how (one or some of) action(s) *y* will satisfy one of corresponding *pro tanto* obligations *e*_*n*_ (note that some values for *e*_*n*_ might be the same as some values for *d*_*n*_): P(*Gw*) = (100/(*ERx* + *ERy*))**ERx* expanded, that is: P(*Gw*) = (100/((*∑*^*n*^*SCa...a*_*n*_**∑*^*n*^*OCd...d*_*n*_) + (*∑*^*n*^*SCb...b*_*n*_**∑*^*n*^*OCe...e*_*n*_)))*(*∑*^*n*^*SCa...a*_*n*_**∑*^*n*^*OCd...d*_*n*_), or if one is ready to assign a single credence to the extended proposition *a* which describes an action as satisfying *pro tanto* oblitations *d...d*_*n*_, and to do the same for *b* concerning *e...e*_*n*_:

$$ \mathrm{P}(Gw)=\left(100/\left(\left({SCa}^{\ast }{\sum}^n OCd\dots {d}_n\right)+\left({SCb}^{\ast }{\sum}^n OCe\dots {e}_n\right)\right)\right)\ast \left({SCa}^{\ast }{\sum}^n OCd\dots {d}_n\right). $$

21 This is equivalent to Swinburne’s suggestion for assigning probabilities to morally equal putative divine actions (2004, 123).

22 See Thurow (2012) for a model which think would helpfully ‘plug in’ to an Anselmian augury in the way I suggest here.
